# Flow Dynamics and Contaminant Transport in Y-Shaped River Channel Confluences

**DOI:** 10.3390/ijerph16040572

**Published:** 2019-02-16

**Authors:** Xiaodong Liu, Lingqi Li, Zulin Hua, Qile Tu, Ting Yang, Yuan Zhang

**Affiliations:** 1 Key Laboratory of Integrated Regulation and Resource Development on Shallow Lake of Ministry of Education, Nanjing 210098, China; 2 College of Environment, Hohai University, Nanjing 210098, China; Lilingqi1995@163.com (L.L.); 15295527223@163.com (Q.T.); yang92005006@163.com (T.Y.); 18262621003@163.com (Y.Z.)

**Keywords:** contaminant transport, flow dynamics, flume model, Y-shaped river channel confluence

## Abstract

River channel confluences are widespread in natural rivers. Understanding their unique hydrodynamic characteristics and contaminant transport rules may facilitate the rational and effective treatment of the water environment. In this study, we considered the Xitiaoxi River Basin as the research area, and a well-designed flume was established based on the extracted water system features. Hydrodynamically, in the Y-shaped confluence channel the flow velocity was easy to separate at the confluence, and a low flow velocity region appeared in the two branches. The spiral flow mainly flowed counterclockwise to the downstream region and the spiral trend increased as the discharge ratio decreased. The spiral flow and its effect on the transport and blending of contaminants were distinct between Y-shaped and asymmetrical river confluences. Based on the flow dynamics test, a set of pollutant discharge devices and a multi-point electrolytic conductivity meter were employed to research the mixing rule for pollutants. A high concentration zone for pollutants was likely to occur near the intersection, and the contaminant concentration band after the confluence was first compressed and then diffused. In particular, line source discharge in the left branch and the point source discharge in the inner bank of the left branch and in the outer bank of the right branch were dominant, and were conducive to the detection and treatment of pollutants.

## 1. Introduction

Channel confluences that are characterized by two channel flows converging into one flow are common in fluvial systems [1]. The main channel and branch branches are connected by junctions to form water systems and even river networks. River networks are important parts of the surface water circulation system and they also represent important channels for watersheds, sediments, and other types of material transported in river basins [2]. At river junctions, the turbulent mixing of flows is severe and there is a strong retention effect on sediment and pollutant transport [3]. Thus, confluences have become a focus area for water conservation, shipping, and environmental protection.

**Figure 1:**
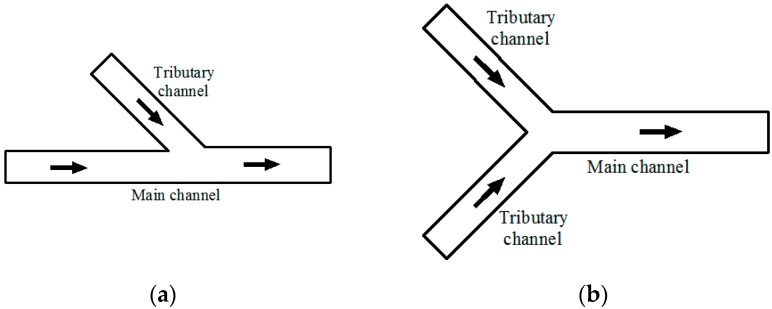
Two types of confluence channels: (**a**) Asymmetrical confluences; (**b**) Y-shaped confluences.

Many types of junctions occur in natural rivers. Junctions can be categorized into either asymmetrical river confluences, where the post-confluence channel forms a linear extension of the upstream main channel, and Y-shaped confluences, where two tributaries are geometrically symmetrical. Both types of intersections are shown in Figure 1.

In addition, there are differences in the hydrodynamic characteristics of the different types of confluences. For example, several studies have investigated asymmetrical junctions, and factors such as confluence ratio and confluence angle are proposed. In particular, Best [4] proposed a model of the flow dynamics present at the river channel confluences based on six characteristic flow zones which include: regions with flow stagnation, flow deflection, flow separation, maximum velocity, downstream flow recovery, and several distinct shear layers. Moreover, Best and Reid [5] conducted four flume experiments with different confluence angles to determine the features of the flow separation zone. The dimension was found to increase with both the junction angle and the contribution of the branch to the total discharge, but the shape remained more or less constant. In addition, a study by Qing et al. [6] indicated that the discharge ratio affects the geometry and separation zone trend from the riverbed up to the surface. Furthermore, Best [7] conducted an in-depth investigation of complex flow and sediment transport patterns, showing that the major controls for these processes are the junction angles and the ratio of the discharge in the two confluence channels.

In addition, the effects of differences in the depth between the main and branch channels have been extensively studied. These studies revealed that depth discordance had the effect of distorting the mixing layer as well as strengthening the turbulence intensity and shear stress between the confluent streams [8,9,10]. Moreover, Hsu et al. [11] proposed a mathematical formulation of the energy and momentum correction coefficients. The study was designed in order to determine the empirical relationship between the momentum transfer and the discharge ratio by studying a 90° equal-width open-channel junction. In a related study, Hsu et al. [12] also showed that increases in the junction angle and downstream Froude number led to an increase in the depth ratio, and they proposed a suitable formula.

The confluence of current also forms unique phenomena such as helical cell and spiral flow. Stream flow analysis was furthered by Weber et al. [13] who determined the three-dimensional flow field within a junction by using an acoustic Doppler velocimeter (ADV). In their report, the velocity vector field, turbulent kinetic energy, and water surface mapping were analyzed. Likewise, Liu et al. [14] also conducted an experimental study of a 90° open channel confluence and found that the vertical distribution of the average velocity was influenced by both the discharge ratio and the flow pattern. Next, Biswal [15] found that the secondary current and turbulent stresses were reproduced well by the hydraulic model, where they increased in the interface region as the relative flow ratio decreased. This was related to a study by Yuan et al. [16] where several hydrodynamic and turbulence characteristics were analyzed. In this study, factors such as the turbulent kinetic energy, Reynolds shear stress, and turbulence spectrum were evaluated to obtain data from a T-shaped discharge-adjustable circulating flume. This experiment was conducted to determine the turbulent flow structure in the distorted shear layer. The results showed that a stronger helical cell was formed and it extended for a longer distance downstream when the branch channel had a higher flow rate than the main channel. In a related study it was found that the pressure gradient term was the primary factor that triggered the velocity redistribution, whereas the convective acceleration was the secondary term as the Froude number increased [17].

The transport of pollutants at channel confluences is influenced by specific hydrodynamic characteristics [18]. Biron et al. [19] used a three-dimensional mathematical model of water flow to conduct numerical simulations in the laboratory, modeling the mixing of pollutants in an open channel. The results showed that the contaminant mixing process was faster when river bed depths were inconsistent between the main channel and tributaries. To address this issue, Isabel et al. [20] established a three-dimensional hydrodynamic model to simulate the effects of contaminant diffusion in the Douro River estuary in Portugal. The results showed that a stable flow was most conducive to the diffusion of contaminants, thereby indicating that the water flow characteristics had important effects on the diffusion of pollutants. In a related study, a two-dimensional model was used to simulate the transport of pollutants under different flow rates at 90° intersections, where the polluted area decreased as the discharge ratio increased [21]. This then affected the concentration of different cross-sectional contaminants according to the distance from the junction point. Based on the Reynolds averaged Navier–Stokes equations and Reynolds stress turbulence model, the distribution of contaminant concentrations is primarily controlled by the shear layer and two counter-rotating helical cells [22]. This phenomenon is affected by the discharge ratio and bed morphology.

However, these previous studies have all considered asymmetrical river confluences, whereas relatively few studies have investigated symmetrical river confluences known as Y-shaped confluences. Guo et al. [23] employed a model to study the three-dimensional hydraulic characteristics of the flows at a Y-shaped junction using data obtained with an acoustic Doppler velocimeter (ADV). According to their results, the stagnation zone, flow deflection zone, flow separation zone, acceleration zone, and other zones could be detected at the “tributary inclined mainstream”-shaped junction. The overall flow was characterized as a spiral flow, which is one of the main properties downstream of a Y-shaped junction. As the discharge ratio became higher and there was bed discordance, the trend of the spiral flow was weakened. Rhoads [24] suggested that helical motion can enhance the mixing patterns at confluences. This was based on observations of the downstream persistence of a well-defined mixing interface at two symmetrical confluences, and the disruption of this interface at asymmetrical confluences. Likewise, Geberemariam [25] suggested that for a 90° junction, the separation zone area and discharge ratio are indirectly proportional due to the recirculating flow, low pressure, and minimum velocities near the T- and Y-junction areas.

Current methods based on the hydrodynamic characteristics of water flows in river intersections can be divided into prototype observational data analyses [26,27], physical model tests [28,29], and numerical simulations [30,31]. Previous studies on Y-shaped river channels mainly focus on hydrodynamic characteristics, but there are few studies on the law of pollutant mixing. In order to address the lack of previous analyses of Y-shaped junctions, flume experiments were performed to investigate the hydrodynamics and contaminant transportation at a 60° channel confluence. We distinguished the differences in the two types of confluences in order to facilitate pollutant management at river confluences.

## 2. Materials and Methods 

**Figure 2:**
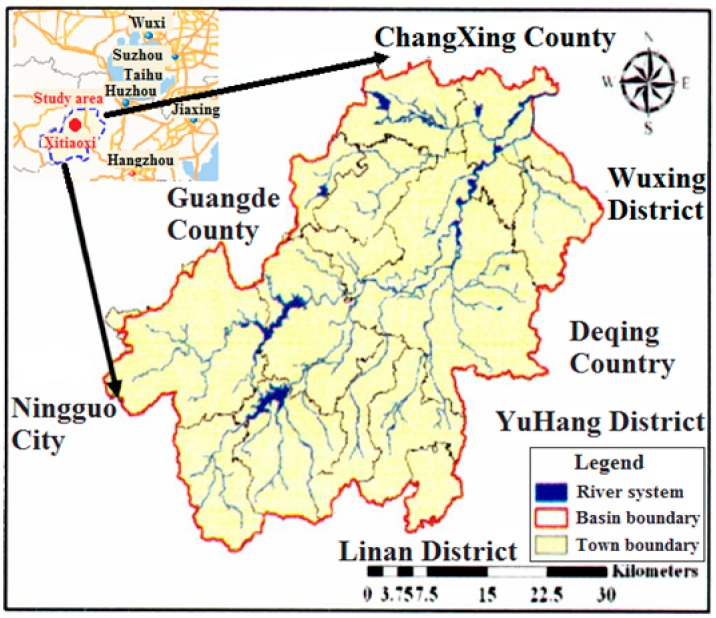
Overview of the study area.

Eight representative channel confluences in Xitiaoxi watershed served as the study sites for the research. The Xitiaoxi river basin, one of the most important tributaries in the Lake Taihu Basin, is a typical dendritic river network (see Figure 2). It is located in Huzhou city, Zhejiang province, and supplies water for residents’ daily living. The classification and morphological analysis of the river network indicate that many confluences exist in Y-shaped form of the Xitiaoxi river basin. Natural confluence river intersection angles ranged between 30° and 90°, with a statistical average of 60°.

In order to ensure that this study had practical significance, the flume model was designed based on the morphological characteristics of the water system in the Xitiaoxi river basin. Table 1 shows the specific data, which indicate that the natural confluence intersection angles ranged between 30° and 90°, with a statistical average of 60°. Thus, the confluence angle employed in the model was 60°.

**Figure 3:**
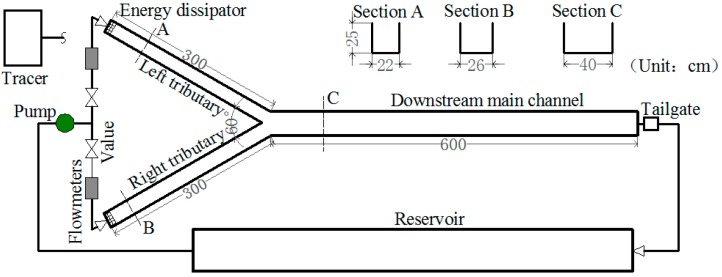
Schematic diagram of the flume model.

A type of flume with a symmetrical confluence was used to perform hydrodynamic disturbance and contaminant transport experiments. The experimental device was designed according to the results of the field investigation. This experiment was a basic investigation where the model and statistics were designed according to a scale of 1:250. According to the gravity similarity criterion, the generalized flat bottom flume fixed bed model was used for the test. The current study is concerned with results of a 60° confluence angel, although the natural confluence river intersection angles ranged between 30° and 90°. The two tributaries were 22 cm and 26 cm wide, respectively, and 3 m long, while the post-confluence channel was 40 cm wide and 6 m long (see Figure 3). It is common for the post-confluence channel to be slightly wider than the branch channel at natural river confluences, a phenomenon referred to as “downstream hydraulic geometry” [32]. This experiment was carried out using a flume that is awaiting national invention patent of China approval (No. 201810287680.8). 

Head tanks on the both branch channels supply the discharge. To ensure a fully developed flow entered into the junction branches, energy dissipator and a sufficiently long channel were placed at the branch channel inlets. Water was pumped from the tank to the two branches through polyvinyl chloride pipes of 110 mm in diameter, and the flow discharges were monitored by two ultrasonic flowmeters and pump-value systems. The water level in the downstream main channel was controlled by an adjustable tailgate (h = 15 cm).

**Figure 4:**
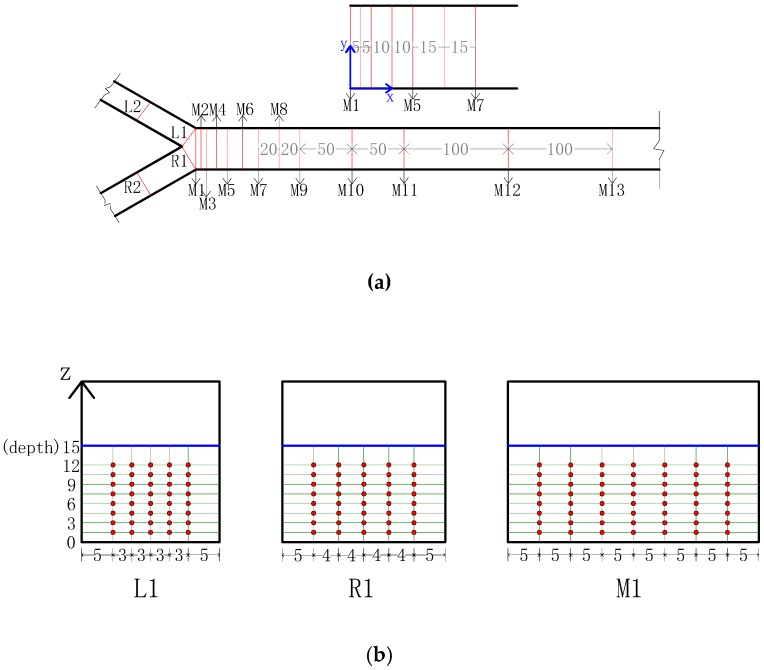
Location of: (**a**) Cross sections; (**b**) Flow velocity measurements.

The coordinate system defined for this testing had the positive x-axis oriented in the downstream direction of the main channel. The positive y-direction points to the left branch wall opposite of the channel junction. Thus, the positive z-axis is upward in the vertical direction. The origin from which all points are measured was the bed at the right branch corner of the channel junction (see Figure 4).

Two discharge ratios are considered in this study. In Case 1 the left branch channel, Q_l_, has a discharge of 10 m^3^/h, and that of the downstream main channel, Q_t_, is 30 m^3^/h, yielding a discharge ratio (q = Q_l_/Q_t_) of 0.33. In Case 2, the left branch channel, Q_l_, the discharge is 20 m^3^/h and for Q_t_ it is 40 m^3^/h, yielding a discharge ratio of 0.5 A combined flow usually comprises a subcritical flow and turbulent flow. Therefore, when designing the flume model, the Froude number was maintained at less than 1 to ensure that the flow was subcritical. The Reynolds number was greater than 1000 to ensure that the flow was turbulent. Within the two discharge ratios mentioned above, the contaminant test used a mixture of sodium chloride, ethanol, and water as the contaminant tracer, which together made the density approximately equal to that of the water body cycled in flume. The tracer was stored in the water tank and discharged into the flume by the tracer discharge device which is plastic tube with discharge hole. The tracer emission manner for the tracer considered both the point source and the line source. According to the different discharge mode, the discharge position was set on different sides of the branch channel. The specific contaminant tracer discharge conditions are shown in Table 2.

According to preliminary studies, the discharge outlets are usually located at half of the water depth in the same direction as the flow of water, which makes the distribution of the emissions more uniform. Therefore, under the different test conditions, the discharge ports were fixed at half of the water depth and the pollutant discharge q_c_ was constant at 0.1 L/s.

A Vectrino acoustic Doppler velocimeter (ADV) was used to measure three-dimensional flow velocities at a series of grid-defined points, which were taken in lines at 13 cross sections (M1–M13), with the near bed locations being more closely spaced as shown in Figure 4a. Each channel cross section consisted of seven evenly spaced vertical profiles, as shown in Figure 4b. In each line, the lowest measurement point is located at 1.5 cm above the bed due to the measuring requirements of the ADV, and other points are located in the line with a vertical interval of 1.5 cm. This testing grid produced approximately 888 velocity measurement locations for each flow condition studied. The velocity measurements were taken at each sampling location for 30 s at a sampling rate of 50 Hz.

In the contaminant transport experiment, a multi-point electrolytic conductivity meter was used to measure the conductivity of the water. This device measured the water conductivity and temperature, and the data were converted into the corresponding pollutant concentrations for further analysis. The sampling data are collected over duration of 180 s, in which approximately 200 instantaneous datums were acquired from each measurement point (average value of the final instantaneous data), thereby ensuring that accurate mean data were obtained.

## 3. Results and Discussion

In order to facilitate data analysis, dimensionless data processing was carried out. For the data processing of geometric dimensions, the x direction (longitudinal direction) and y direction (transverse direction) were dimensionless, with a width of 40 cm of the downstream main channel. In the z direction (vertical direction), the design depth at the tail gate was dimensionless. All the results of this experiment were dimensionless.

### 3.1. Flow Dynamics at Channel Confluences Transport of Contaminants at Channel Confluences

#### 3.1.1. Longitudinal Velocity

**Figure 5:**
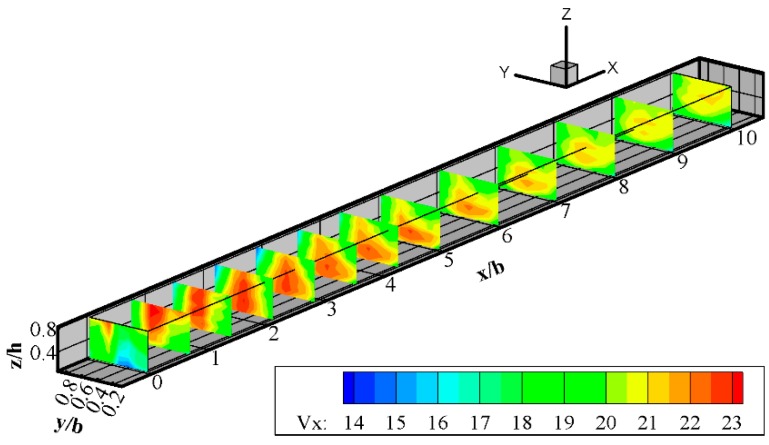
Contour plot showing the longitudinal flow velocities after the intersection (Qr = 0.5).

The longitudinal velocity v_x_ is the dimension velocity in the x-axis measured in cm/s. Figure 5 displays longitudinal velocity contours along the downstream main channel for q = 0.5. The flow velocities were relatively large in the middle and lower layers, whereas they were relatively small in the near surface layer as a result of the lateral momentum being greater near the surface than near the bed. In this study, several basic flow characteristics can be found from the distribution of velocity components in the cross sections. The low velocity zone can be seen as the area of relatively lower velocity along the junction-adjacent wall downstream of the channel junction. Adjacent to the low velocity zone is the velocity acceleration zone resulting from the contraction of the two confluent flows by the low velocity zone. The maximum velocity can reach about 23.46 cm/s and this occurs just downstream of the junction at x/h = 2.5. At the interface of the two incoming flows is the shear layer, which is characterized by a velocity gradient caused by the velocity difference between the two incoming flows.

All longitudinal velocity contours near the bed are distinctly different from the near surface velocity patterns. The low velocity zone is larger near the left bank—both in length and width its size varies from top to bottom—because of the different dimensions of the same flow of two branches channels. The higher momentum for the small section area condition allows the left branch channel flow to extend further into the main channel before being deflected downstream, therefore causing a wider low velocity zone. In the constricted reach immediately downstream of the junction, higher velocities occur near the bed. However, once the contracted region is passed, velocities readjust to the typical open-channel condition of higher velocities near the surface. As can be seen from Figure 5, the flow did not completely recover from the junction effect until beyond x/h = 10.

#### 3.1.2. Secondary Current and Two Helical Cells

**Figure 6:**
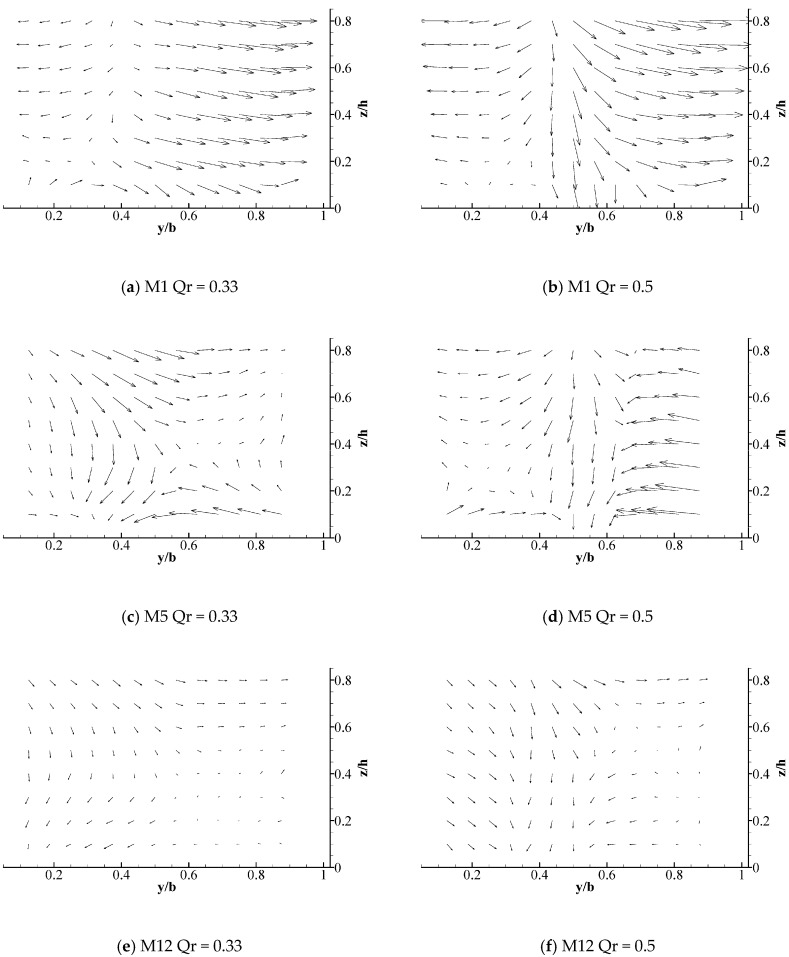
Longitudinal flow velocity distribution and secondary flow structure in the mainstream measurement section.

Two secondary currents in the opposite direction, which are initiated by the deflection of the symmetric tributaries collision, can be found in junction cross-section 1 (see Figure 6). The border between two symmetric secondary current can be observed at y/h = 0.45. The secondary current is gradually weakened along the downstream channel. Up to M12, the secondary current tends to disappear and the velocity becomes uniform. In addition, when the discharge ratio is larger, the flows in the left and right bank have a more uniform distribution, and the secondary current becomes more evident. A greater momentum with a larger discharge ratio in two branches results in a remarkable secondary current. This was due to the conflicting flow caused by the branches, and the two boundary conditions made the fluid produce two relatively symmetrical flows that deviated from the main flow direction.

**Figure 7:**
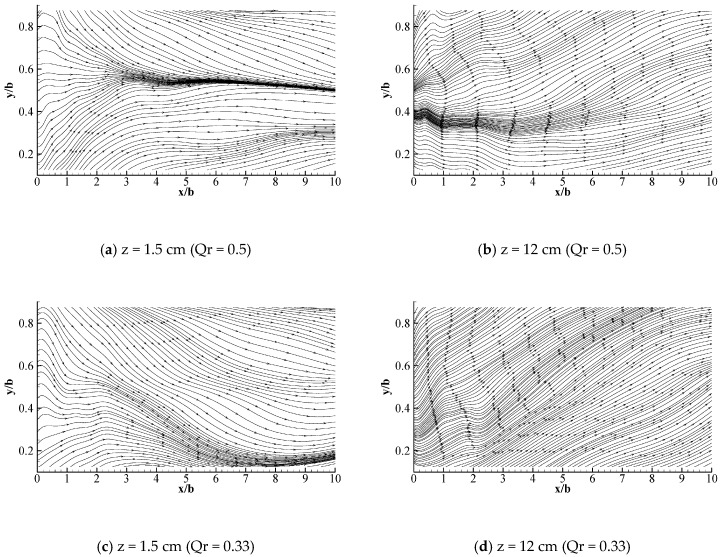
Longitudinal and horizontal streamline diagrams for the intersecting water flows.

**Figure 8:**
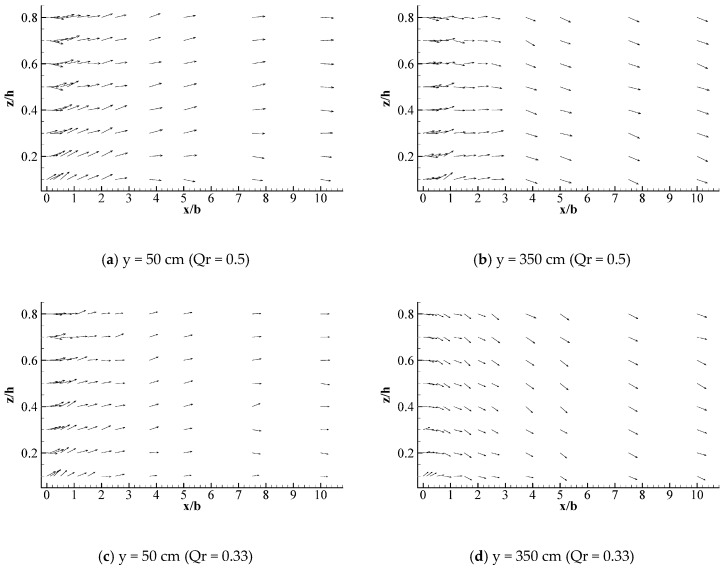
Horizontal flow velocity vector for the intersecting water flow.

Figure 7a shows that the two water flow deflection zones are symmetrical when the discharge ratio is 0.5, which is consistent with the characteristics of the Y-shaped junction channel. After the intersection, the overall water flow deviation at z/h = 0.1 is contrary to that at z/h = 0.8, where the upper water flow is biased to the left bank while the lower water flow is biased to the right bank (see Figure 7). Figure 8 demonstrates that the water near the left bank flows downward, whereas the water flow near the right bank flowed upward. Therefore, the Y-shaped intersecting water flow comprised a double-spiral flow at the intersection, which was a counter-clockwise vortex toward the downstream. The rotation intensity decayed as the distance departing from the junction increased and the discharge ratio decreased.

Two helical cells with large momentum appeared after the junctions. Under the action of the inertial force, the upper water flow was partial to the left side while leaving the right side hollow. However, continuous flow brought about the water flow with a small momentum at the bottom portion, penetrating into the right side by the action of the gravitational potential and the pressure potential energy. This is the biggest difference between Y-shaped confluences and asymmetrical confluences in terms of their hydrodynamic characteristics. Similar characteristics of spiral flow towards the downstream region in a Y-shaped junction were obtained in previous studies based on the hydraulic characteristics of water flows in 90° Y-shaped intersections [23]. Nonetheless, the dimensions of the low velocity zone and the vortex trends of the overall spiral flow generated by the 60° Y-shaped intersection were smaller than those with a 90°confluence angle.

### 3.2. Transport of Contaminants at Channel Confluences

#### 3.2.1. Different Discharge Manners and Locations

**Figure 9:**
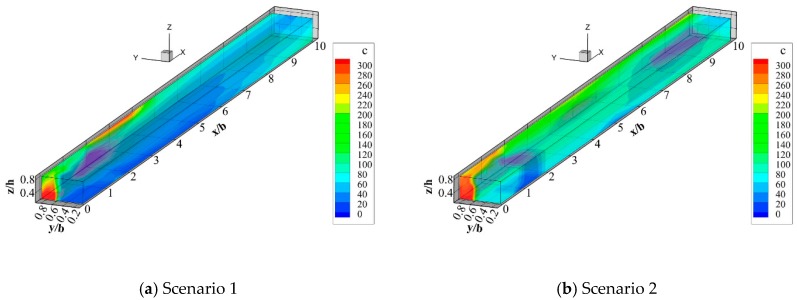

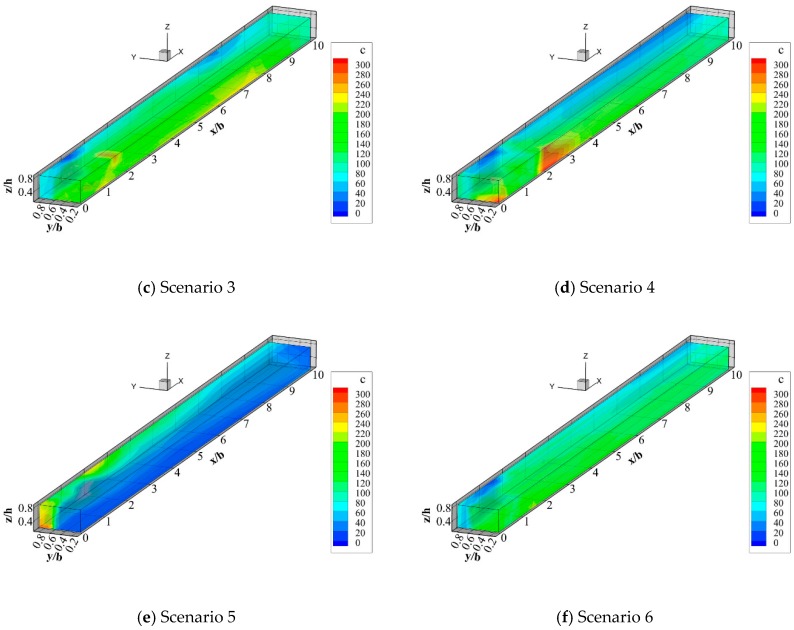
Three-dimensional distribution maps of point source emission concentration fields (Qr = 0.33).

Figure 9 depicts the distributions of contaminant concentrations at sections along the longitudinal direction in different discharge locations. Apparently, the distributions of contaminants are distinctive due to diverse flow characteristics for different scenarios. In all scenarios, there was a bending band with a large gradient of contaminant concentration at the interface between the polluted and clean water, which is named the mixing layer. A concentration partition at the intersection with a high concentration area adjacent to the left branch and a low concentration area close to the right branch can be found in Figure 9a. The pollutants were then spread downstream until they were evenly mixed. By comparing Figure 9a,b, the point source discharge position on the left inner bank could shrink the half length and width of the high-concentration pollution zone in contrast with that on the outer bank. The polluted water discharged at the left outer side was easier to pollute in the downstream channel, which can be attributed to the flow diffusion along main channel caused by the relatively low pressure in the low velocity zone. Thus, the location of the left branch point source for discharging pollutants facilitated the blending of the pollutants at the inner bank. Similarly, Figure 9c shows that when pollutants are discharged by point source at the right branch, a large concentration area formed along downstream near the right branch whereas a low concentration area near the left branch. Figure 9c,d demonstrates that when the pollutant discharged by the point source on the outer bank of the right branch, the length of the high-concentration pollution zone is shorter than that when the discharge pollutant on the inner bank. The maximum concentration zone appeared at the junction and downstream close to the junction when the contaminant was released at the outer bank. By contrast, the maximum concentration area appeared downstream of the junction when the concentration was discharged at the inner bank. Therefore, the point source discharge of pollutants on the right branch side facilitates the mixing of the pollutants better compared with the point source discharge on the inner bank, which means that it is more conducive to the river training.

The distributions of contaminant concentrations when the pollution tracer is discharged from the line source are shown in Figure 9e,f. The water can also be separated into three parts, including the mixing layer at the interface between the clean and polluted water, the clean water, and the polluted water. When the discharge come from a line source, the contaminant concentration band after the intersection is first compressed and then diffused downstream, where the pollutant concentration region is twisted. The line source discharge from the left branch full-section produces a high concentration area near the left branch side of the mainstream after the intersection while discharge from right generates a low concentration area near the left branch side along the downstream area of the mainstream. The maximum concentration value shown in Figure 9e is greater than that in Figure 9f, but the overall concentration in Figure 9e is lower than that in Figure 9f, i.e., the maximum concentration of pollutants discharged from the left branch by line source is higher than that from the right one. However, the overall concentration becomes lower after mixing, which is more conducive to the transport and blending of contaminants.

The phenomenon where the contaminant concentration band was located after the convergence compressed initially was due to the top support action of each branch, and then the diffusion was mainly a result of the secondary current in the confluence. Moreover, the Y-shaped intersecting water tended to rotate counter-clockwise toward the downstream, so the pollutant concentration zone was twisted. The high concentration zone is likely to appear at the intersection and downstream near the intersection. Combining the velocity distribution law, these regions are often low velocity zones or even flow separation zones. On account of the sudden decrease in the velocity to almost zero, the pollutants are stagnant and the maximum concentration zone appears. Some pollutants mix rapidly in the high velocity zone and a low concentration zone appears in the maximum velocity zone.

#### 3.2.2. Different Discharge Ratios

**Figure 10:**
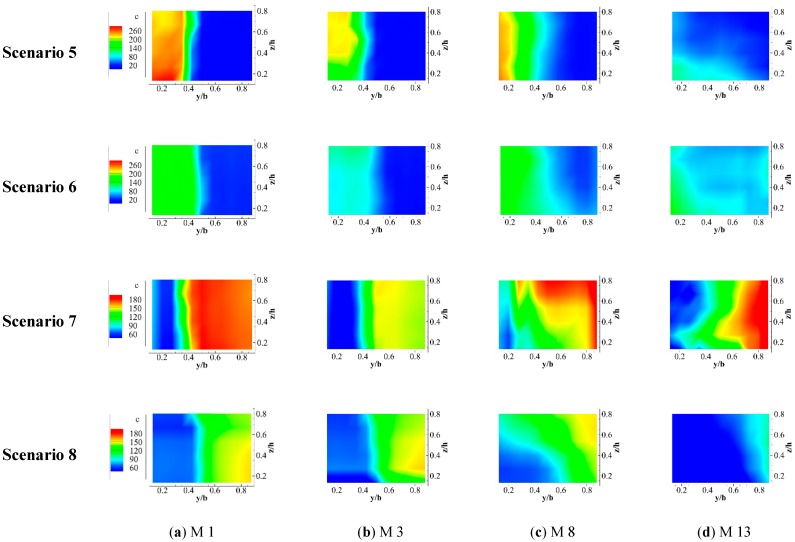
Concentration diagram for the pollutant discharge from the left branch line source under different convergence ratios.

In Scenarios 5 and 6, a high pollution concentration zone appears near the left bank at M1 (see Figure 10). The contaminant zone, which contains a mixing layer and polluted water, is larger than that where q = 0.33, but the maximum concentration value is smaller. However, all the features are reversed when pollutant tracer is discharged by line source at the right branch. The high concentration pollution zone occurs close to the right bank at the confluence and downstream. The range of the contaminant zone and the maximum concentration value when q = 0.33 are both larger than those when q = 0.5. As the discharge ratio increases, with the constant discharge mode and emissions, the left branch’s flow and momentum augment due to the smaller of cross section. At M3, the maximum concentration of the contaminant band disappears and the concentration is redistributed because the water flows interchange and blend with each other after the intersection. At M8, the high concentration contaminant zone reappears and it blends into the center downshift due to the counter-secondary flow (see Figure 8). The pollutants are basically mixed up evenly by M13. Therefore, when contaminant is discharged from the left branch, the identification and treatment of pollutants will be better in the small discharge ratio. On the contrary, when the confluence ratio is larger, the identification and treatment of pollutants will be better when the discharge of pollutants is from the right branch.

## 4. Conclusions

According to the morphological characteristics and similarity theory for the Xitiaoxi water system as a typical dendritic river network area, as well as the actual size of the test site, a physical model test system was developed for a 60° Y-type intersection in order to analyze the hydrodynamic characteristics. Furthermore, pollutant discharge devices and multi-point conductivity meters were employed in pollutant blending tests. Several conclusions can be drawn based as follows. 

(1) The water flow in the 60° Y-shaped confluence was vulnerable to flow velocity separation at the junction, thereby forming a small-range low-flow region located in the two branches downstream of the water flow intersection. After the intersection, the flow direction moved downward near the left side, whereas the flow direction was upward near the right bank.

(2) A double-spiral flow with equivalent strength appeared at the intersection and a spiral flow with a counter-clockwise vortex flow was present downstream. The vortex intensity decreased with the downward flow of the water and the rotation trend of the spiral flow decreased as the discharge ratio increased.

(3) The contaminant concentration band that appeared after the confluence tended to be compressed and it then diffused downstream, where it was generally twisted. A high concentration pollutant zone is likely to occur at the junction and downstream near the junction.

(4) The discharge of pollutants from the point source on the inner bank on the left branch was more conducive to the transport and mixing of the contaminants than that from the outer bank on the left branch, and the concentration was lower after mixing evenly, whereas the opposite was found on the right branch.

(5) The discharge was higher from the line source on the left branch than that from the right branch. A higher concentration contaminated area was readily produced near the junction, but the overall concentration was lower after mixing, which is more beneficial for the transport and blending of contaminants. When the pollutants were released from the left branch, the discharge ratio was smaller, the pollution belt width was smaller, and the mixing was more rapid and effective. This was more favorable for the identification and treatment of pollutants, but the opposite was found for the right branch.

The spiral flow and its effect on the transport and blending of contaminants were the main differences between Y-shaped and asymmetrical river confluences. The results obtained for 60° and 90° Y-shaped intersections were similar, but the size of the separation zone at 60° and the vortex tendency of the overall spiral flow were reduced as compared to at 90°.

## 5. Patents

This experiment was carried out using a flume that is awaiting national invention patent of China approval (No. 201810287680.8).

## Figures and Tables

**Table 1:** Width and confluence angle of the river at the Y-shaped confluence channels.

Y-shaped Confluence	Left Branch Mean Width (m)	Right Branch Mean Width (m)	Mainstream Mean Width (m)	Confluence Angle (°)
**1**	100.21	107.81	198.81	58.59
**2**	69.06	80.15	106.15	48.35
**3**	27.81	30.83	54.65	30.81
**4**	98.23	100.88	163.15	60.98
**5**	54.14	73.18	100.88	69.27
**6**	33.31	48.02	54.15	75.03
**7**	32.61	38.17	44.63	92.73
**8**	29.56	40.30	74.14	45.57
**Average value**	55.62	64.92	99.57	60.17

Notes: “Left” and “right” denote the directions of the downstream flow.

**Table 2:** The specific contaminant tracer discharge conditions.

Scenario	Discharge (m³/h)		Contaminant Discharge Methods	Contaminant Discharge Position
	Left branch	Right branch		
1	10	20	point source	The outer bank of the left branch
2	10	20	point source	The inner bank of the left branch
3	10	20	point source	The outer bank of the left branch
4	10	20	point source	The inner bank of the left branch
5	10	20	line source	The full section of left branch
6	10	20	line source	The full section of right branch
7	20	20	line source	The full section of left branch
8	20	20	line source	The full section of right branch

Notes: The inner bank of branch denotes the bank near the intersection. The branch flow units are all m^3^/h.
